# A Peculiar Form of Traumatic Conjunctivitis

**Published:** 1905-12

**Authors:** 


### A Peculiar Form of Traumatic Conjunctivitis.

A. M. HUTTON contributes: Some miners employed in sinking a shaft near here (Navarre, Mich.) encountered numerous streams of sulphur water. Though a careful analysis of the water has not been made it is sufficient for me to state that it gives rise to an acute conjunctivitis. The pain is most excruciating, and can be relieved only by the use of cocaine, and even cocaine is useless unless preceded by adrenalin chloride.

My practice has been to use adrenalin chloride, 1-2000, and to follow this with cocaine, 2 per cent. solution, and then to give the patient a boracic-acid-and-cocaine solution to be used until all symptoms have disappeared.

The point in favor of adrenalin chloride is this: Cocaine will *not* relieve this condition, unless preceded by adrenalin chloride.

